# Divergent Strategy in Marine Tetracyclic Meroterpenoids Synthesis

**DOI:** 10.3390/md19050273

**Published:** 2021-05-13

**Authors:** Antonio Rosales Martínez, Ignacio Rodríguez-García, Josefa L. López-Martínez

**Affiliations:** 1 Department of Chemical Engineering, Escuela Politécnica Superior, University of Sevilla, 41011 Sevilla, Spain; 2 Organic Chemistry, ceiA3, University of Almería, 04120 Almeria, Spain; irodrigu@ual.es (I.R.-G.); pepaloma91@hotmail.com (J.L.L.-M.)

**Keywords:** divergent total synthesis, marine natural products, tetracyclic meroterpenoids, aureol

## Abstract

The divergent total synthesis strategy can be successfully applied to the preparation of families of natural products using a common late-stage pluripotent intermediate. This approach is a powerful tool in organic synthesis as it offers opportunities for the efficient preparation of structurally related compounds. This article reviews the synthesis of the marine natural product aureol, as well as its use as a common intermediate in the divergent synthesis of other marine natural and non-natural tetracyclic meroterpenoids.

## 1. Introduction

**Figure 1:**
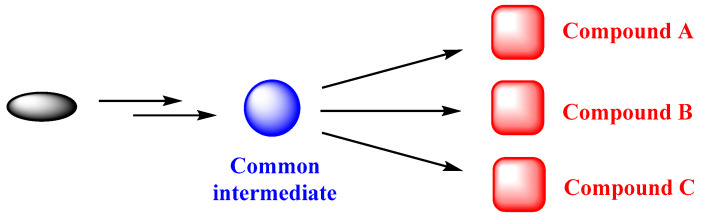
Divergent total synthesis.

The original definition of divergent total synthesis (Figure 1) was reported by Boger et al. [1] and was defined as the synthesis in which “at least two members of the class of compounds” can be separately prepared from a common, advanced synthetic intermediate. Therefore, the most important challenge in a divergent synthesis is the choice of a common intermediate which could be transformed into a target array of natural products and non-natural derivatives. This strategy is a powerful tool that has attracted the attention of numerous research groups as it improves the efficiency of chemical processes [2,3], and attains special relevance when structure-activity studies are the ultimate goals. Later, other terms such as “diverted total synthesis” [4] and “collective total synthesis” [5] were introduced, thus extending the amplitude of divergent synthesis. In this way, “diverted total synthesis” can be applied to the preparation of a natural product-like compound library by appropriate transformations of a common intermediate, avoiding the limitations inherent to partial syntheses from the natural product caused by the presence of multiple similar functional groups. In addition, the term “collective total synthesis” is used when the common intermediate is endowed with functional characteristics suitable for the preparation of structurally diverse natural products belonging to different families.

**Scheme 1:**
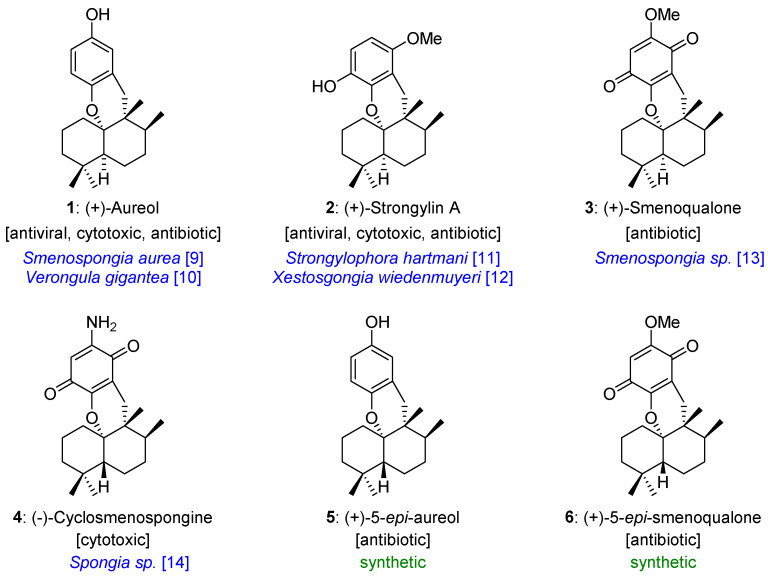
Representative examples of marine natural and non-natural tetracyclic meroterpenoids and their reported biological activity.

On the other hand, tetracyclic meroterpenoids [6,7] are a unique class of marine natural compounds with significant biological activities. Representative examples of marine natural and non-natural tetracyclic meroterpenoids, including (+)-aureol (**1**) [8,9,10], (+)-strongylin A (**2**) [8,11,12], (+)-smenoqualone (**3**) [8,13], (−)-cyclosmenospongine (**4**) [8,14], (+)-5-*epi*-aureol (**5**) [8,15,16,17], and (+)-5-*epi*-smenoqualone (**6**) [8] have been considered of interest by the chemical community due to their interesting biological properties and unique molecular structures (Scheme 1). In fact, structure–activity relationship (SAR) studies show that variations on the nature and substituents on the aromatic ring have a strong impact on the observed activity [8,18]. These natural products (Scheme 1) contain a compact tetracyclic system with a substituted benzopyran moiety, four consecutive asymmetric carbon atoms, and a well-defined *trans*- or *cis*-relationship between the two cyclohexane rings of the decalin system. Although several synthetic methods have been described, a divergent approach to this class of compounds has not been previously reported as such.

**Scheme 2:**
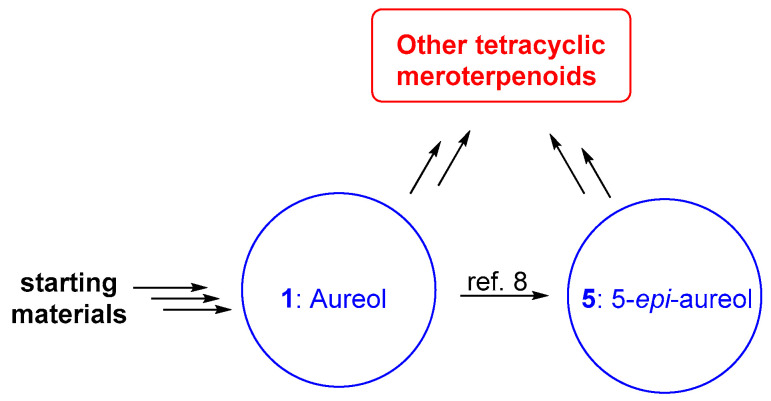
Conceptual model of the divergent synthesis of tetracyclic meroterpenoids using aureol (**1**) as a pluripotential late-stage intermediate.

This article focuses on the synthetic efforts towards aureol (**1**), a marine natural meroterpenoid present in the Caribbean sponges *Smenospongia aurea* [9] and *Verongula gigantea* [10] which has shown an important biological profile [8,19,20]. Aureol (**1**) can be an excellent advanced and common synthetic intermediate for the divergent synthesis of other natural and non-natural tetracyclic terpenoids. In this article, we present a unified and versatile approach for the diversification of this class of compounds with the aim to contribute to the development of new desirable drugs for the pharmaceutical industry and the medicinal chemistry. The divergent synthesis of either natural or fully synthetic derivatives could be achieved through aureol (**1**) as a common intermediate, by adequate sequential functionalization of the aromatic ring, or by epimerization of the decalin core of aureol (**1**) to 5-*epi*-aureol (**5**) followed by functionalization of the aromatic ring (Scheme 2).

**Scheme 3:**
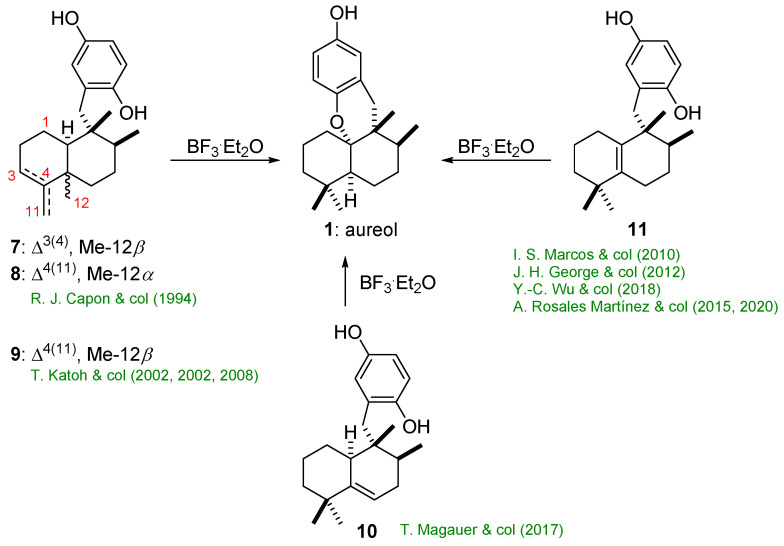
Key olefinic intermediates of previous syntheses of aureol (**1**).

Scheme 3 summarizes the last step of previous syntheses towards aureol (**1**). All of them have as key step a cationic cyclization of an olefinic intermediate (**7**–**11**).

The different synthetic sequences for aureol (**1**) are listed below, classified according to the olefinic key intermediate shown in Scheme 3.

## 2. Synthesis of Aureol

### 2.1. Synthesis of Aureol from Key Intermediates ***7***–***9***

#### 2.1.1. Capon’s Synthesis of (+)-Aureol

**Scheme 4:**
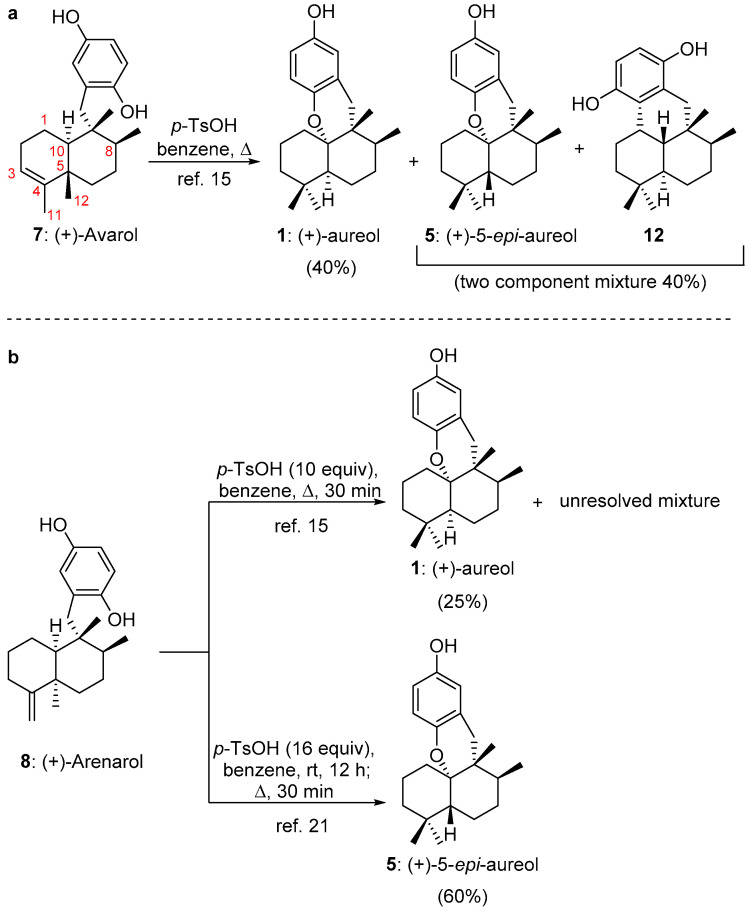
(**a**): Acid-induced rearrangement of (+)-avarol (**7**). (**b**): Acid-induced rearrangement of (+)-arenarol (**8**).

The first work on the synthesis of the marine product (+)-aureol (**1**) was published by the group of R. J. Capon [15] using natural sesquiterpene hydroquinones ((+)-avarol (**7**) and (+)-arenarol (**8**)) as starting materials (Scheme 4). In these processes (+)-aureol (**1**) could be formed via a concerted 1,2-migration of Me-12 and H-10. However, formation of (+)-*epi*-aureol (**5**) is better understood considering that, after methyl migration, there is a loss of the C-10 proton to give a Δ^5,10^ olefin intermediate, which would later suffer *trans* addition of the OH group. This lack of stereocontrol of the process was later confirmed by Lakshmi et al. [21], as they could determine the structure of **5** by X-ray analysis (Scheme 4b).

#### 2.1.2. Katoh’s Synthesis of (+)-Aureol

**Scheme 5:**
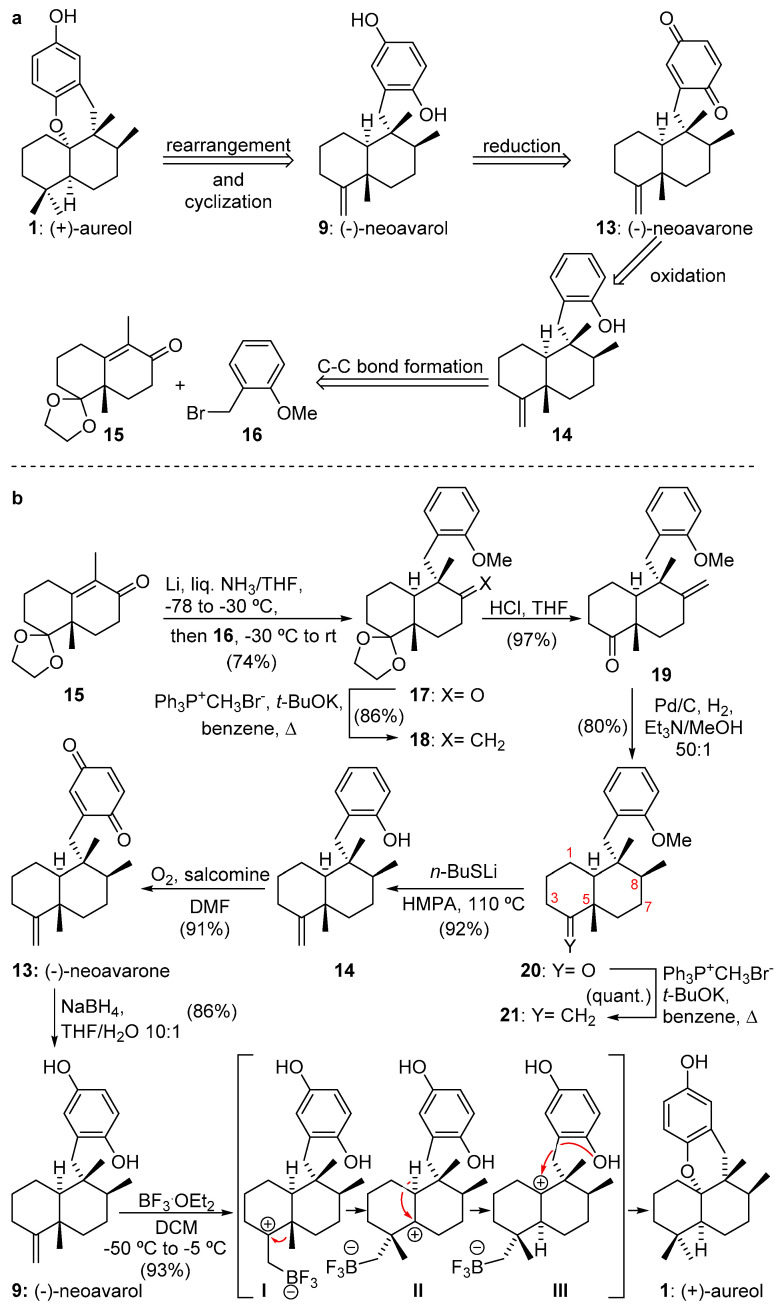
Strategy for the synthesis of (+)-aureol (**1**) according to Katoh and colleagues [22,23,24]. (**a**): Retrosynthetic plan. (**b**): Synthesis of (+)-aureol (**1**). HMPA = hexamethylphosphoramide; salcomine = *N,N*′-bis(salicylidene)ethylenediamino cobalt(II); DMF = *N,N*′-dimethylformamide.

Katoh and colleagues [22,23,24] reported the first enantioselective total synthesis of (+)-aureol (**1**) in 2002 [22], a process they later improved in 2003 [23]. The retrosynthetic plan of the improved synthesis is shown in Scheme 5a. This approach obtains aureol (**1**) in one step by acid-induced rearrangement/cyclization of (−)-neoavarol (**9**). In turn, **9** can be prepared by reduction of the quinone moiety present in (−)-neovarone (**13**), a compound which can be readily obtained by strategic salcomine oxidation of **14**. This can be assembled by stereocontrolled reductive alkylation of (+)-5-methyl-Wieland-Miescher ketone (**15**) with 2-methoxybenzyl bromide (**16**) (Scheme 5a).

As shown in Scheme 5b, Katoh’s synthesis of (+)-aureol (**1**) used enantiopure (+)-5-methyl-Wieland-Miescher ketone (**15)** as the starting material. A C-C bond-forming reaction between **15** and a lithiated arene unit, prepared from 2-methoxybenzyl bromide (**16**), gave the coupling product **17** as a single diastereomer in 74%. The Wittig methylenation of **17** produced the *exo*-double bond present in decaline **18** in 86% yield. Removal of the acetal protective group by acid treatment (97% yield), followed by hydrogenation of the exocyclic double bond present in the resulting ketone **19**, led to the product **20** (80% yield) together with its C8 epimer (13% yield). Subsequent Wittig methylenation of **20** quantitatively gave **21**, which was submitted to a deprotection of the *O*-methyl group in order to form the phenol **14** (92% yield). O_2_/salcomine oxidation of **14** gave the quinone **13** (91% yield). Finally, NaBH_4_ reduction of quinone **13** gave (−)-neoavarol (**9**) (86% yield). Once **9** was synthesized, the crucial step was the BF_3_^.^Et_2_O-induced rearrangement of **9,** which led to the desired (+)-aureol (**1**) (93% yield). This rearrangement occurred via stereospecific 1,2-hydride and methyl shifts, as shown in Scheme 5b. In this reaction the three intermediate carbocations **I**, **II**, and **III** are involved. This synthesis was completed in nine steps (33% overall yield) from the starting material **15**.

### 2.2. Synthesis of Aureol from Key Intermediate ***10***

#### Magauer’s Synthesis of (+)-Aureol

**Scheme 6:**
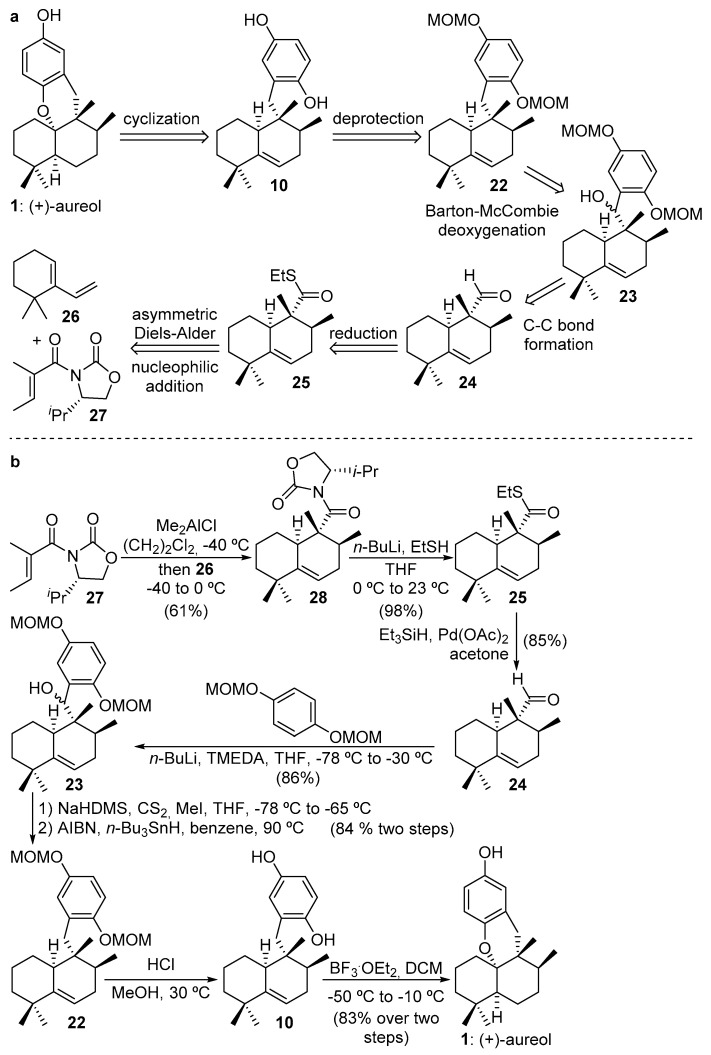
Strategy for the synthesis of (+)-aureol (**1**) according to Magauer et al. [8]. (**a**): Retrosynthetic plan. (**b**): Synthesis of (+)-aureol (**1**). TMEDA = *N,N,N′,N*′-tetramethylethane-1,2-diamine; NaHDMS = sodium bis(trimethylsilyl)amide; AIBN = 2,2′-azobis(2-methylpropionitrile).

The synthesis of (+)-aureol (**1**) reported by Magauer and colleagues [8] used a highly robust and modular synthetic platform, developed for the preparation of natural and fully synthetic analogues of tetracyclic meroterpenoids. The retrosynthetic plan for (+)-aureol (**1**) (Scheme 6a) is based on the stereospecific acid-promoted cyclization of the Δ^5(6)^ olefin intermediate **10**. Its methoxymethyl ether derivative **22** could be prepared from **23** following the Barton–McCombie deoxygenation protocol. This diastereoisomeric mixture of benzyl alcohols results from the addition of 2-lithiohydroquinone dimethyl ether to aldehyde **24**, which can be obtained by Fukuyama’s reduction [25] of thioester **25**.

The keystone in the asymmetric process is the formation of the three chiral centers present in **25** through the asymmetric Diels–Alder reaction [26,27] between **26** and enantiopure **27** followed by lithium ethanethiolate removal of the chiral auxiliar (Scheme 6a).

Scheme 6b details the synthesis of (+)-aureol (**1**) from diene **26**. The first key step was the asymmetric construction of the 5,6-dehydrodecaline component **28** employing an *exo*-selective Diels–Alder cycloaddition between diene **26** and tiglic acid-derived dienophile **27** to afford **28** (61% yield). The oxazolidinone chiral auxiliar was replaced by nucleophilic 1,2-addition of lithium ethanethiolate to the carbonyl group of the Diels–Alder product **28**, a process which afforded thioester **25** in 98% yield. A smooth Fukuyama reduction [25] of **25** gave aldehyde **24** (85% yield), which was coupled with the lithiated arene unit to afford a mixture of diastereomeric benzylic alcohols **23** (86% yield). The free hydroxy group of **23** was removed using the two-step Barton–McCombie deoxygenation protocol, which afforded **22** in 84% yield (two steps). The subsequent deprotection of **22** with HCl/MeOH gave hydroquinone sesquiterpene **10**, which was directly subjected to cyclization conditions to give (+)-aureol (**1**) (83% yield). In this reaction, the proton formed by coordination of BF_3_^.^OEt_2_ to one of the OH-groups in **10** possibly triggers the cationic rearrangement. When the cyclization is carried out under kinetic conditions (at temperatures below −10 °C), a *cis*-decaline framework is formed exclusively. On the other hand, under thermodynamic control, only the *trans*-decaline is obtained. This total synthesis was achieved in eight steps (30% overall yield) from the starting material **26**.

### 2.3. Synthesis of Aureol from Key Intermediate ***11***

#### 2.3.1. Marcos’s Synthesis of (−)-Aureol

**Scheme 7:**
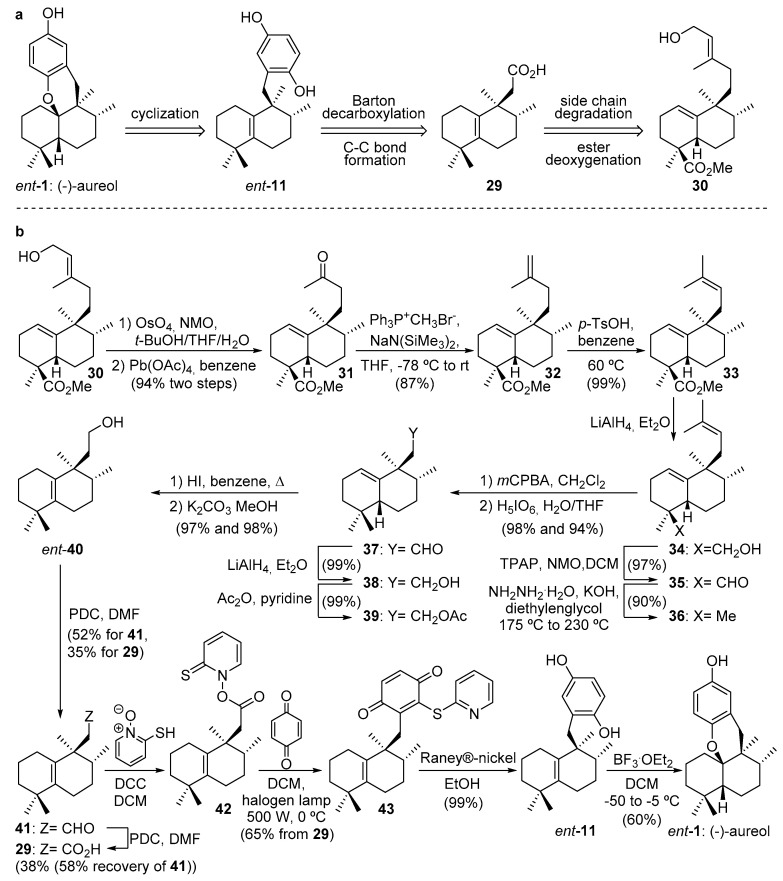
Strategy for the synthesis of (−)-aureol (*ent*-**1**) according to Marcos and colleagues [26]. (**a**): Retrosynthetic plan. (**b**): Synthesis of (−)-aureol (*ent*-**1**). NMO = *N*-methylmorpholine-*N*-oxide; TPAP = tetra-*n*-propylammonium perruthenate; *m*CPBA = *m*-chloroperoxybenzoic acid; PDC = pyridinium dichromate; DCC = *N,N*′-dicyclohexylcarbodiimide.

Marcos and colleagues [28] reported the total synthesis of the (−) enantiomer of aureol (*ent*-**1**) from the methyl ester of natural *ent*-halimic acid (Scheme 7a). Their approach was based on: (a) the acid-induced cyclization of sesquiterpene hydroquinone *ent*-**11**, (b) the Barton decarboxylation reaction/*p*-benzoquinone addition sequence and the subsequent reduction with Raney^®^ nickel (*ent*-**11** from **29**), and (c) the side-chain degradation of *ent*-halimic acid methyl ester **30** and the subsequent reduction of C-18 methyl ester.

As shown in Scheme 7b the synthesis of (−)-aureol (*ent*-**1**) used *ent*-halimic acid methyl ester **30** as the starting material. The degradation of the side chain of **30** was achieved [29,30] by oxidation with OsO_4_ followed by Pb(OAc)_4_, which gave ketone **31** (94% yield, two steps). The synthesis of the *endo*-olefin **33** required the Wittig methylenation of **31** (87% yield) and subsequent acid isomerization of **32** (99% yield). In order to remove the C-18 methyl ester, a three steps sequence from **33** to **36** was used, a process which gave a very good global yield. The synthesis of product **39** was achieved in four steps: a) the chemoselective epoxidation of the side-chain double bond in **36** (98% yield), b) the oxidative cleavage with H_5_IO_6_ in H_2_O/THF to afford **37** (94% yield); c) reduction with LiAlH_4_ (99% yield) to give **38** (99% yield), and d) the acetylation of the hydroxy group in **38** to afford **39** (99% yield). The isomerization of the olefin double bond present in acetate **39** with HI (97% yield) followed by the saponification of the acetoxy group (98% yield) gave the rearranged product *ent*-**40**. Finally, the oxidation of *ent*-**40** to acid **29** via aldehyde **41** was achieved with pyridinium dichromate (PDC) in a moderate yield. Once intermediate **29** was available, the key precursor *ent*-**11** of (−)-aureol (*ent*-**1**) could be readily prepared by Barton decarboxylation reaction in the presence of *p*-benzoquinone, a methodology reported by Theodorakis and colleagues [31,32] for the synthesis of ilimaquinone. In this way, when **29** was treated with 2-mercaptopyridine *N*-oxide in the presence of *N,N*′-dicyclohexylcarbodiimide (DCC) a photo labile thio-hydroxamic ester (**42**) was obtained. Then, **43** was prepared by light-induced decarboxylation (halogen lamp 500W) of **42** in the presence of benzoquinone in a 65% yield from **29**. The subsequent reduction of **43** with Raney^®^ nickel gave *ent*-**11** in a 99% yield. With the key precursor *ent*-**11** in their hands, the treatment of this compound with BF_3_^.^Et_2_O at low temperature exclusively afforded (−)-aureol (*ent*-**1**) with complete stereoselectivity (60% yield). This total synthesis was achieved in 19 steps (10.3% overall yield) from *ent*-halimic acid methyl ester **30** as the chiral pool starting material.

#### 2.3.2. George’s Synthesis of (+)-Aureol

**Scheme 8:**
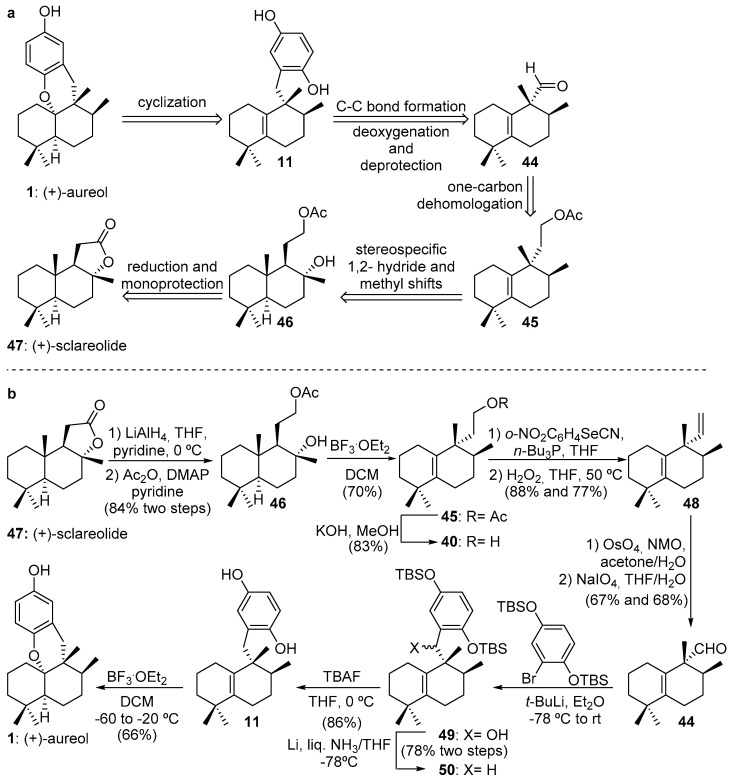
Strategy for the synthesis of (+)-aureol (**1**) according to George and colleagues [33]. (**a**) Retrosynthetic plan. (**b**) Synthesis of (+)-aureol (**1**). DMAP = 4-(dimethylamino)pyridine; NMO= *N*-methylmorpholine-*N*-oxide; TBAF = tetrabutylammonium fluoride.

George’s group [33] published in 2012 the second total synthesis of (+)-aureol (**1**). Their biosynthetically inspired retrosynthesis of (+)-aureol (**1**) (Scheme 8a) rests upon the biomimetic acid-mediated cyclization of the key tetrasubstituted olefin intermediate **11**, which could be prepared through a process involving the addition of an aryllithium derivative to aldehyde **44**. This aldehyde could be formed using a one-carbon dehomologation sequence from **45**. Another key step in the process is the biomimetic sequence of 1,2-hydride and 1,2-methyl shifts, which converts alcohol **46** into **45**. Finally, the reduction and selective protection of the commercially available enantiopure starting material (+)-sclareolide (**47**) would form the intermediate **46**.

As shown in Scheme 8b, George’s synthesis of (+)-aureol (1) used **11** as the key intermediate, which was prepared from natural (+)-sclareolide (**47**). Its reduction with LiAlH_4_ gave a diol, which was selectively protected at the primary hydroxy group with Ac_2_O in pyridine to afford the acetate **46** in an 84% yield (two steps). Monoacetate **46** was stereoselectively converted to the single stereoisomer olefin **45** (70% yield) in a rearrangement induced by BF_3_^.^Et_2_O, which occurred via stereospecific sequential 1,2-hydride and 1,2-methyl shifts. Saponification of the acetate in **45** gave alcohol **40** (83% yield), which was readily converted into aldehyde **44** through a one-carbon dehomologation sequence using the Grieco–Sharples elimination protocol [34,35] (67% yield in two steps) followed by oxidative cleavage of the resulting terminal alkene **48** (45% yield in two steps). With aldehyde **44** in their hands, the coupling between **44** and an aryllithium species gave the mixture of diastereomeric benzylic alcohols **49**. In order to remove the OH group, this mixture of alcohols (**49**) was treated with lithium in liquid ammonia followed by NH_4_Cl aqueous solution to afford deoxygenated compound **50** in a 78% yield (two steps). Removal of the TBS protecting groups in **50** with tetrabutylammonium fluoride provided the key intermediate **11** in an 86% yield. To complete the synthesis of (+)-aureol (**1**), the intermediate **11** was treated with BF_3_^.^Et_2_O to afford (+)-aureol (**1**) in a 66% yield. This total synthesis was achieved in 12 steps (6% overall yield) from (+)-sclareolide (**47**).

#### 2.3.3. Wu’s Synthesis of (+)-Aureol

**Scheme 9:**
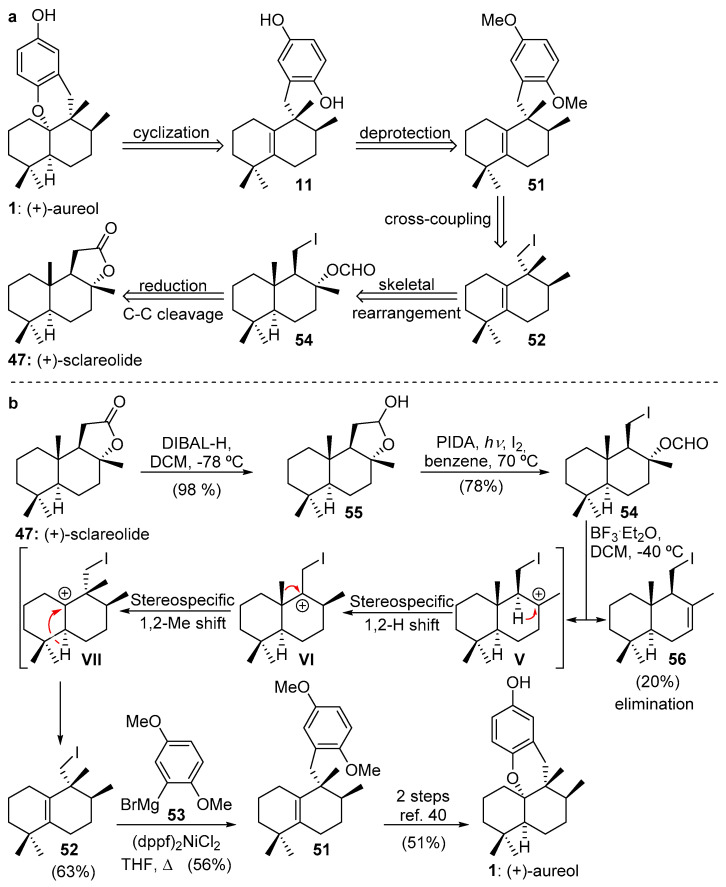
Strategy for the synthesis of (+)-aureol (**1**) according to Wu and colleagues [36]. (**a**): Retrosynthetic plan. (**b**): Synthesis of (+)-aureol (**1**). DIBAL-H = diisobutylaluminium hydride; PIDA = (diacetoxyiodo)benzene.

In 2018, Wu and colleagues [36] published the formal synthesis of (+)-aureol (**1**). Their retrosynthesis of (+)-aureol is outlined in Scheme 9a. This retrosynthetic analysis is based on: (a) the biomimetic acid-mediated cyclization of the hydroquinone **11** to generate (+)-aureol (**1**), (b) the removal of the two O-Me protecting groups of **51** to afford the key intermediate **11**, (c) the cross-coupling reaction between alkyl iodide **52** and Grignard reagent **53** to give the intermediate **51**, (d) the rearrangement reaction of **54** to afford **52**, and (e) the reduction of (+)-sclareolide (**47**) and subsequent C-C bond cleavage to give **54**.

As shown in Scheme 9b, the synthesis of intermediate **11** was carried out starting from commercially available (+)-sclareolide (**47**). Reduction of **47** using diisobutylaluminium hydride (DIBAL-H) generated sclareal **55** in a 98% yield. The treatment of **55** under the C-C bond cleavage conditions described by Suárez and colleagues [37] gave drimanal iodoformate (**54**) in a 78% yield. The crucial step was the BF_3_^.^Et_2_O-mediated rearrangement of **54**, which occurred via stereospecific sequential 1,2-hydride and 1,2-methyl shifts to generate the desired alkyl iodide **52** (63%), together with a minor amount of by-product **56** (20%). In this reaction, the intermediate carbocations **V**-**VII** could be involved. With alkyl iodide **52** in their hands, the cross-coupling reaction between Grignard reagent **53** and alkyl iodide **52** generated the key intermediate **51** in a 56% yield. As olefin **51** was an advanced intermediate in the Rosales’s synthesis [38,39] of (±)-aureol (**1**), their strategy constituted a formal synthesis of (+)-aureol (**1**). This formal synthesis was completed in four steps (27% overall yield) from starting material (+)-sclareolide (**47**).

#### 2.3.4. Rosales Martínez’s Synthesis of (±)-Aureol

**Scheme 10:**
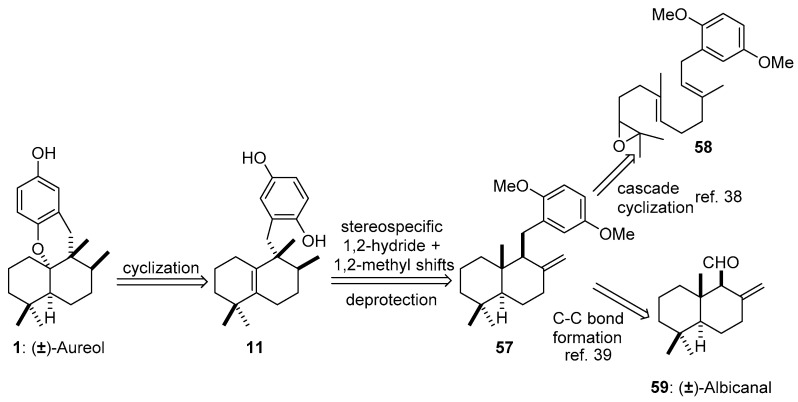
Retrosynthetic plan of (±)-aureol (**1**) according to Rosales Martínez et al. [38,39].

As a part of our efforts directed towards the synthesis of marine terpenoids [40], we embarked on a project aimed at the divergent synthesis of tetracyclic meroterpenoids. Our endeavors started with the racemic preparation of (±)-aureol (**1**) in 2015 [38], a process we latter improved in 2020 [39]. This effort continues with the divergent synthesis of other tetracyclic meroterpenoids using aureol (**1**) as a common synthetic intermediate. The retrosynthetic plan for each synthesis is shown in Scheme 10. Our strategy is based on the preparation of (±)-aureol (**1**) through the biomimetic acid cyclization of hydroquinone **11**, an intermediate that could be generated from **57** through a sequence of 1,2-hydride and 1,2-methyl shifts and the subsequent deprotection of both O-Me groups. **57** is an intermediate common to both synthetic approaches. In one of them, **57** is prepared through Cp_2_TiCl-catalyzed reductive epoxide cyclization cascade of epoxyfarnesol derivative **58** and the subsequent deoxygenation of the OH-group. In the other, a cross-coupling reaction between albicanal (**59**) and 2-lithiohydroquinone is used.

**Scheme 11:**
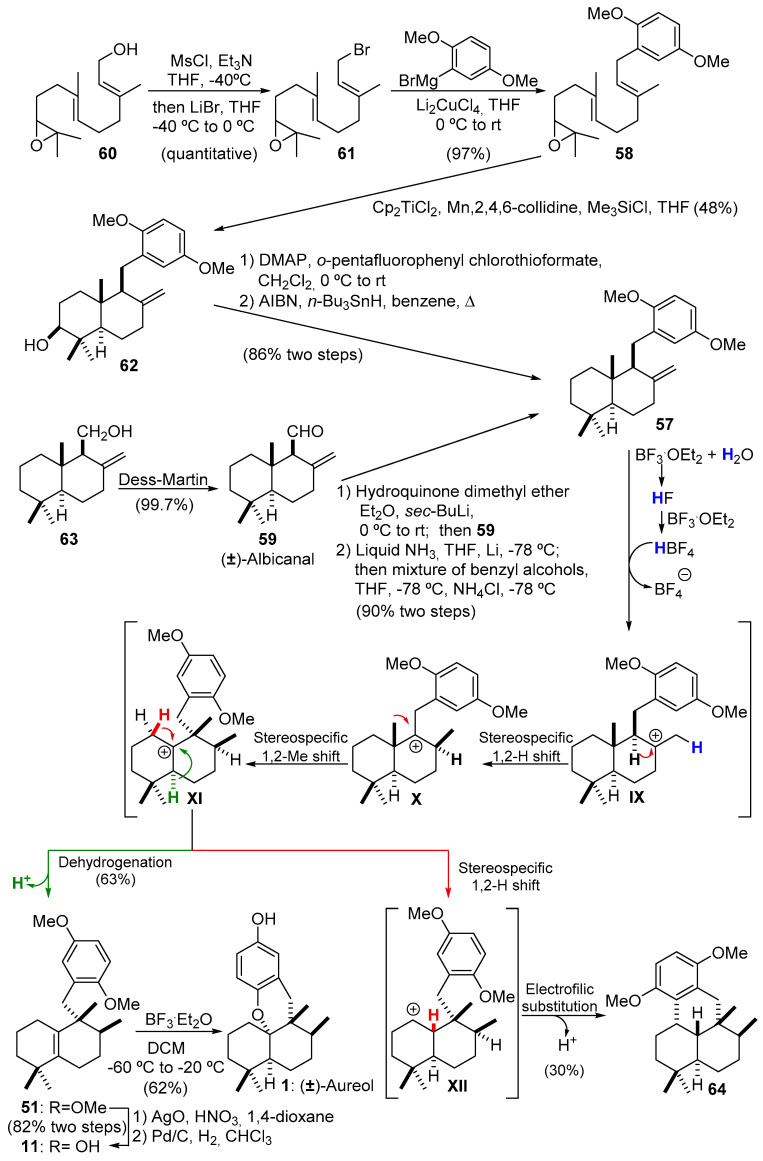
Synthesis of (±)-aureol (**1**) according to Rosales Martínez et al. [38,39]. DMAP = 4-(dimethylamino)pyridine; AIBN = 2,2′-azobis(2-methylpropionitrile).

Initially [38], we pursued the synthesis of the key intermediate **57** using epoxyfarnesol **60** as the starting material (Scheme 11). The one-pot mesylation of product **60** with MsCl, and the subsequent addition of LiBr quantitatively gave a yield of bromide **61**. The cross-coupling reaction between **61** and 2,5-dimethoxyphenylmagnesium bromide afforded the epoxyfarnesol derivative **58** (97% yield). A very elegant Cp_2_TiCl-catalyzed [40] radical cascade cyclization of **58** gave **62** in a moderate 48% yield. The subsequent deoxygenation of alcohol **62** was carried out using the Barton–McCombie procedure, which afforded **57** in an 86% overall yield (two steps). Later [39], the key intermediate **57** was also prepared through a C-C bond-forming reaction between 2-lithiohydroquinone dimethyl ether and (±)-albicanal (**59**) as starting material, which was previously obtained by oxidation of (±)-albicanol (**63**) with the Dess–Martin reagent (99.7% yield). In this way, the coupling of **59** with 2-lithiohydroquinone dimethyl ether gave a mixture of diastereomeric benzylic alcohols which, without separation, was treated with lithium in liquid NH_3_/THF followed by aqueous NH_4_Cl to give the deoxygenated product **57** in a 90% yield (two steps). With **57** in our hands, tetrasubstituted olefin **51** was synthesized by biomimetic-type rearrangement of **57** mediated by BF_3_^.^Et_2_O.

Under these conditions, **51** was obtained in a 63% yield, together with the by-product **64** in a 30% yield. In this reaction, the intermediate carbocations **IX**-**XI** could be involved [39]. The cationic rearrangement might be initiated by a proton from HF, which could be formed through hydrolysis of BF_3_, since is known that BF_3_^.^Et_2_O is very moisture sensitive. The demethylation of **51** gave **11** in an 82% yield over the two steps. Finally, the treatment of the hydroquinone **11** with BF_3_^.^Et_2_O afforded aureol (**1**) (62%). This cyclization was originally explored by Marcos et al. [28] This synthesis of racemic (±)-aureol (**1**) was completed in eight steps (14% overall yield) from the starting material epoxyfarnesol (**60**) or in seven steps (28% yield overall yield) from the starting material (±)-albicanal (**59**).

## 3. Aureol as Pluripotent Late-Stage Intermediate for the Synthesis of Tetracyclic Meroterpenoids

**Scheme 12:**
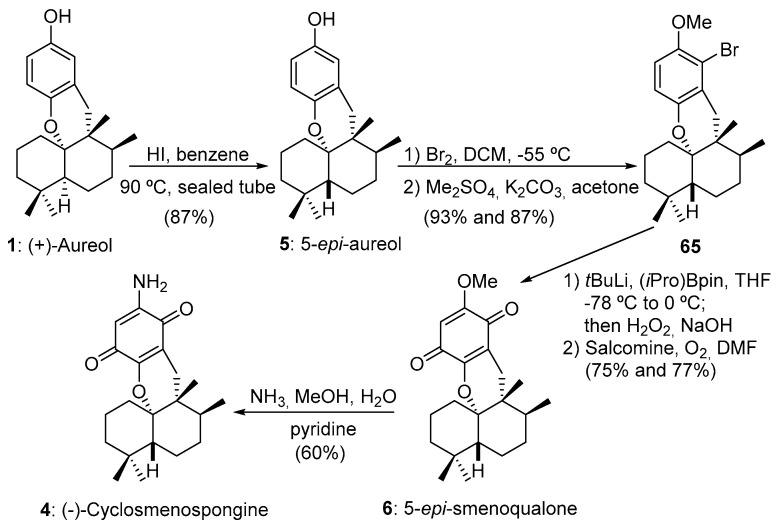
Synthesis of (-)-cyclomenospongine (**4**) and 5-*epi*-smenoquealone (**6**) from (+)-aureol (**1**). DMF = *N,N*′-dimethylformamide.

The possibility of using aureol (**1**) as a late-intermediate for the divergent synthesis of other tetracyclic terpenoids stems from the fact that these compounds have differences mostly on the aromatic moiety. Furthermore, the easy epimerization of aureol (**1**) into 5-*ep*i-aureol (**5**) previously described by Magauer and colleagues [8] opens the door to the preparation of *trans*-decaline. From both *cis*- or *trans*-decaline frameworks, it should be quite straightforward the access to a library of natural or non-natural tetracyclic meroterpenoid analogues, just by simple variation of the arene moiety. The examples represented in Scheme 12 illustrate how other compounds can be obtained from aureol (**1**). The non-natural 5-*epi*-aureol (**5**) was synthesized by thermal isomerization of (+)-aureol (**1**) using hydroiodic acid in benzene at 90 °C (87% yield) [8]. From 5-*epi*-aureol (**5**), the compounds (−)-cyclosmenospongine (**4**) and 5-*epi*-smenoqualone (**6**) were prepared by sequential functionalization of their aromatic core. In this way, selective bromination of **5** with Br_2,_ and the subsequent methylation gave the compound **65** in an excellent yield. The non-natural 5-*epi*-smenoqualone (**6**) was prepared from **65** via a boronation-oxidation sequence in a 58% yield (two steps). Eventually, non-natural **6** was converted to (−)-cyclomenospongine (**4**) via aminolysis (60% yield). In addition, the application of this sequential functionalization of the aromatic core to (+)-aureol (**1**) could be used to prepare natural (+)-smenoqualone (**4**).

## 4. Conclusions

The divergent synthesis is a valuable tool in the design of efficient routes for the synthesis of natural products using a common intermediate. Although several unified strategies have been reported for some families of natural products, it is desirable to extrapolate this methodology to the synthesis of tetracyclic meroterpenoids. In this context, this article reviews the synthesis of the marine natural product aureol (**1**), with special emphasis on their strategies and methodologies. In addition, this natural tetracyclic meroterpenoid can be used as pluripotent late-stage intermediate for the synthesis of other natural and non-natural tetracyclic meroterpenoids. In this article, we proposed a methodology based on a diversification strategy that we believe will be useful in future research for the preparation of other tetracyclic meroterpenoids as substances that could be used as new drugs or in structure–activity relationship studies.

## Data Availability

Not applicable.
